# Attitude Determination System for a Cubesat Experiencing Eclipse

**DOI:** 10.3390/s23208549

**Published:** 2023-10-18

**Authors:** Kesaobaka Mmopelwa, Teddy Tumisang Ramodimo, Oduetse Matsebe, Bokamoso Basutli

**Affiliations:** 1 Department of Mechanical, Energy, and Industrial Engineering, Fauculty of Engineering, Botswana International University of Science and Technology, Private Bag 16, Palapye 10071, Botswana; 2 Department of Electrical, Computer, and Telecommunications Engineering, Fauculty of Engineering, Botswana International University of Science and Technology, Private Bag 16, Palapye 10071, Botswana

**Keywords:** attitude estimation, expectation maximization, cubesat, extended Kalman filtering, attitude kinematics

## Abstract

In the context of Kalman filters, the predicted error covariance matrix $P_{k+1}$ and measurement noise covariance matrix R are used to represent the uncertainty of state variables and measurement noise, respectively. However, in real-world situations, these matrices may vary with time due to measurement faults. To address this issue in CubeSat attitude estimation, an adaptive extended Kalman filter has been proposed that can dynamically estimate the predicted error covariance matrix and measurement noise covariance matrix using an expectation-maximization approach. Simulation experiments have shown that this algorithm outperforms existing methods in terms of attitude estimation accuracy, particularly in sunlit and shadowed phases of the orbit, with the same filtering parameters and initial conditions.

## 1. Introduction

Cube satellites (CubeSats) are being developed because they are capable of carrying out missions such as Earth observation, astronomical physics, etc., in place of large satellites [1,2]. Additionally, CubeSats, which are predominant in low Earth orbit (LEO), have been considered to be key enablers in global coverage and connectivity [3]. Furthermore, they use commercial off the shelf (COTS) components, which reduces development and production costs. A CubeSat is a standard cubic satellite with a side of 10 cm and a mass of approximately 1 kg. Large CubeSats can be built using several cubic units, for instance, two-, three- and six-unit CubeSats. CubeSats have increased access to space by providing low launch cost since they are a carried as secondary payloads in the launch vehicle. Although they are relatively cheap to produce and launch, they have limitations in size, weight, and power (SWaP), limiting the size and performance of the subsystems [4].

**Figure 1:**
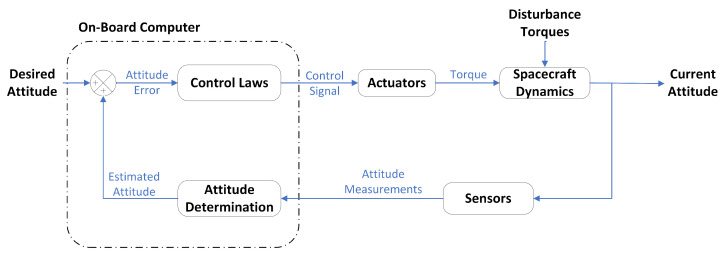
Attitude determination and control system.

The attitude determination and control system (ADCS) is one of the satellite subsystems, and its key function is centered around steering the spacecraft to its intended orientation. The ADCS can further be divided into two subsystems: attitude determination and attitude control subsystems. The former uses sensors to compute the rotation of a rigid body about its center of mass (attitude), while the latter uses actuators to reorient the rigid body to the desired rotation. The desired orientation could be nadir pointing to help ground stations to telecommand or sun pointing to maximize power generation. It is evident in Figure 1 that the attitude determination system (ADS) serves as a reference for control.

The ADS sensors are broadly categorized into reference and inertial sensors. The reference sensors provide directional measurements of the satellite regarding another celestial object in space, for instance, horizon sensors, sun sensors, etc. [4]. Inertial sensors are used to determine the rate of attitude change of an object about an inertial frame of reference [5]. These sensors typically include gyroscopes, which are used to measure angular velocity. Since the ADS uses COTS components, the sensors are usually categorized by high noise levels, which affect the systems’ accuracy. The accuracy of CubeSat attitude estimation can also be influenced by the accumulated attitude error over time, which is caused by drifts of the inertial sensors used.

The attitude determination or estimation algorithms are used to process sensor measurements to compute the attitude. The computed attitude can take several parameterizations; for instance, it may be described using Euler angles, quaternions, axis angle or directional cosine matrix (DCM) [6,7,8]. However, minimal parameterizations such as Euler angles, Rodriguez parameters and modified Rodriguez parameters (MRPs) are frequently avoided in filter designs for global attitude because they can fall victim to singularities, but they are commonly used to define local error attitudes [9]. The most popular attitude parameterization technique is the quaternion since it encounters no singularity problem.

The attitude determination algorithms or single frame methods use measurement vectors provided by reference sensors to calculate an object’s orientation instantaneously. These deterministic algorithms include triaxial attitude determination (TRIAD), Davenport’s q method, quaternion estimator (QUEST) and single value decomposition (SVD) [10,11]. These deterministic methods have been well defined in [12]; therefore, we will not go into detail here. Attitude estimation algorithms make use of vector measurements, inertial measurements and previous attitude information to provide the current optimal attitude estimate. The estimation process can be divided into filtering of sensor measurements and computation of attitude from sensor observations [13]. As stated in [13,14], the filtering process can be accomplished through various methods, such as particle filtering and derivatives of the Kalman filter.

It is common to couple a magnetometer and a sun sensor to measure attitude in small satellites such as CubeSat because each sensor cannot provide three-axis attitude knowledge independently [15,16,17,18,19]. When one vector is available, the satellite is free to rotate about that vector; thus, at least two vectors are used [15]. When the satellite is in orbit, it passes through regions where the sunlight is blocked by the Earth, referred to as the eclipse phase. For low Earth orbit satellites, they spend about 35 min of their orbital time in this phase [20]. During this region, only one vector is available for computing the satellite’s attitude, resulting in high attitude error. The high attitude error affects attitude control accuracy, and this may result in loss of communication during overpasses. It is possible to use additional auxiliary sensors during this phase, but due to the CubeSat’s limited mass and volume, only minimal components are used.

In an attempt to make the attitude estimation process accurate during both the sun and eclipse phases, several methods have been employed. The authors in [21] propose the use of magnetic field derivatives as the second vector in the QUEST-aided multiplicative EKF architecture to increase the accuracy of attitude estimation during the eclipse phase. Using only magnetometer measurements, the proposed method can estimate rough attitude and angular rate even during eclipse phases; therefore, it can also be used in case of gyroscope failure. In [22], it was found that the SVD-aided EKF outperformed the traditional EKF during the eclipse phase. This improved performance was due to the capability of the SVD-aided EKF to adapt the measurement error covariance matrix (R) when the ADS lost sensor measurements. During the eclipse phase, when the satellite was in shadow and the reference sensors might not have been providing reliable measurements, the SVD-aided EKF utilized an adaptation rule to update the R matrix. This adaptation allowed the filter to effectively account for the loss of sensor measurements and to adjust the covariance matrix accordingly, leading to improved attitude estimation accuracy. This adaptive behavior of the SVD-aided EKF during the eclipse phase was found to be a significant advantage over the traditional EKF, which did not incorporate such an adaptation. As a result, the SVD-aided EKF demonstrated superior performance in terms of attitude estimation accuracy during this challenging phase of the satellite’s orbit, as shown in the research findings by [22]. In contrast, the research revealed that the SVD-aided EKF may exhibit poor performance over a longer period (e.g., over 1000 s) compared to the traditional EKF, particularly during extended eclipse phases. For instance, in the case of low Earth orbit (LEO) CubeSats, the eclipse phase typically lasts for approximately 35 min (2100 s), as shown in [20]. As a result, the SVD-aided EKF may not be well suited for attitude estimation during such prolonged eclipse phases. Another paper [23] proposes a prediction approach for estimating satellite attitude during the eclipse phase and an adaptive scheme that utilizes the SVD-aided EKF to determine the sun phase. However, the study identified that the accuracy of the prediction algorithm was compromised due to the prolonged duration of the eclipse period.

The SVD-aided EKF was developed as an attempt to enhance the performance of the traditional EKF by incorporating measurement noise covariance matrix adaptation. However, it has been observed that the SVD-aided EKF may face limitations in prolonged eclipse phases. In response, further research has been conducted to develop improved approaches that can make the EKF more adaptable to anomalies such as measurement faults during extended eclipse periods. In [24], the authors present a technique for computing satellite attitude estimation using an EKF with singular value decomposition (SVD) assistance. The algorithm also incorporates simultaneous adjustments of the process and measurement covariance utilizing data obtained from magnetometers and sun sensors. When the process noise increases or the spacecraft is in eclipse, SVD-aided adaptive EKF performs better than SVD-aided EKF. The authors in [25] propose a hierarchical and efficient framework for satellite attitude determination that aims to compensate for observation errors in raw attitude data. It includes a simplified adaptive Kalman filtering module, a neural-network-based system error compensation module, and a weighted attitude smoothing module. The performance of the proposed framework, trained with full matrix elements, was found to be comparable to the optimal accuracy when compared with conventional algorithms. Another study in [26] introduces a novel algorithm called adaptive iterated extended Kalman filter (AIEKF) for relative position and attitude estimation, considering model uncertainty in a nonlinear stochastic discrete-time system with unknown disturbance. The AIEKF algorithm employs Gauss–Newton iterative optimization steps for maximum a posteriori (MAP) estimation and incorporates a switch-mode combination technique to achieve adaptability. The mean-square estimation error (MSE) of the state estimate is derived, and it is proven that the AIEKF outperforms the traditional extended Kalman filter (EKF) or iterated extended Kalman filter (IEKF) in terms of MSE.

The structure of the paper is as follows: The attitude estimation and static algorithms are presented in Section 2 in detail. Section 3 provides an introduction to the mathematical models utilized for the presented CubeSat. The proposed filter adaptation method is presented in Section 4. In Section 5, the simulation results of the adaptive EKF algorithm for a hypothetical CubeSat are presented. In Section 6, a brief conclusion is given.

## 2. Attitude Estimation Algorithms

In this section, we look at the singular value decomposition, extended Kalman filter and their hybrid, as they have been proven to be robust algorithms.

### 2.1. The SVD Algorithm

The SVD algorithm is a deterministic technique for computing the attitude matrix that minimizes Wahba’s problem [11,27]. An alternative way to simplify the Wahba’s loss function is to use a matrix B given as:

(1) $$B=\sum_{k=1}^{N}w_{k}b_{k}r_{k}^{T}={\mathrm{USV}}^{T},$$

where $w_{k}$ is the non-negative weight, $r_{k}$ is a set of reference vectors in the reference frame, and $b_{k}$ is a set of measured vectors in the satellite body frame. The optimal attitude matrix is calculated using U and V matrices, such that

(2) $$A_{opt}=U\mathrm{diag}\left[\begin{array}{ccc} 1 & 1 & \det(U)\det(V) \end{array}\right]V^{T}.$$

To examine the rotation angle error, the error covariance matrix is defined as

(3) $$P_{svd}=U\mathrm{diag}{\left[(s_{2}+s_{3}\right)}^{-1}\;{\left(s_{3}+s_{1}\right)}^{-1}\;{\left(s_{1}+s_{2}\right)}^{-1}],$$

where $s_{1}=S_{11}$, $s_{2}=S_{22}$ and $s_{3}=\det U\det VS_{33}$.

### 2.2. The Extended Kalman Filter

The extended Kalman filter (EKF) is appropriate for nonlinear state equations; therefore, it can be used as an alternative attitude estimator [28]. Nonlinear discrete time systems can be defined as follows:

(4) $$x_{k}=f\left(x_{k-1},w_{k},k\right),$$

(5) $$z_{k}=h\left(x_{k+1},k\right)$$

where state vector x is involved in the system and measurement differential functions denoted by *f* and *h*, respectively, *y* is the expected measurement, and *w* and *v* are the process and measurement noises with known covariance, respectively. The EKF consists of two steps: prediction and measurement update, that is,

*Prediction step*

(6) $$x_{k+1}=F_{k}x_{k-1},$$

(7) $$P_{k+1}=F_{k}P_{k-1}F_{k}^{T}+Q_{k}.$$

*Measurement Update*

(8) $$K_{k}=\frac{P_{k+1}H_{k}^{T}}{H_{k}P_{k+1}H_{k}^{T}+R_{k}},$$

(9) $${\hat{x}}_{k}=x_{k+1}+K_{k}\left(z_{k}-z_{k}\right)$$

(10) $$P_{k}=P_{k+1}-K_{k}H_{k}P_{k+1}.$$

The differential function *h* is deduced from the relationship between reference vectors r and the satellite measured vectors, b given by

(11) b=Ar,

where A is the attitude matrix in quaternion form. The measured vector b is the expected measurement given a set of references vector; therefore, it is similar to $z_{k}$ from (5).

### 2.3. SVD-Aided EKF Algorithm

The SVD-aided EKF reduces the computational cost of the EKF by using rough attitude estimates from the SVD algorithm instead of non-linear measurements [24]. The estimation covariance obtained from the SVD is used as the measurement noise covariance matrix in the filter, which inherently makes it adaptive to noise increments in measurements. In the SVD-aided EKF, the discrete time system is defined as

(12) $$x_{k+1}=f\left[x_{k},k\right]+w_{k}$$

(13) $$z_{k}=Gx_{k+1}+v_{k}.$$

## 3. System Model

### 3.1. System Kinematics

The change in attitude of a satellite is modeled using the kinematics equation, which describes rotational motion despite the cause. The kinematic equation of a satellite using quaternions attitude representation is given by

(14) $$\dot{q}=\left[\begin{array}{c} \dot{q_{0}}\\ \dot{q_{1}}\\ \dot{q_{2}}\\ \dot{q_{3}} \end{array}\right]=\frac{1}{2}\left[\begin{array}{ccc} -q_{1} & -q_{2} & -q_{3}\\ q_{0} & -q_{3} & q_{2}\\ q_{3} & q_{0} & -q_{1}\\ -q_{2} & q_{1} & q_{0} \end{array}\right]\left[\begin{array}{c} p\\ q\\ r \end{array}\right]$$

where p,q and *r* are the components of the angular velocity ω provided by the gyroscope [29], and $q_{0}$ is the scalar component of the quaternion. The kinematic equation is useful when defining the state transition matrix in the extended Kalman filter. In this research, quaternions are employed for attitude computation due to their mathematical advantages, such as singularity avoidance and efficient numerical representation. However, for graphical presentation and visualization, Euler angles are used, as they are more commonly used in aerospace engineering and provide a more intuitive representation of attitude. This choice allows for a clear and easy-to-understand presentation of the results in a familiar format. The computed quaternion is converted to Euler angles using

(15) $$\begin{array}{cc} \; & \phi =(2\left(q_{0}q_{1}+q_{2}q_{3}\right),1-2\left(q_{1}^{2}+q_{2}^{2}\right)),\\ \; & \theta =(2\left(q_{0}q_{2}-q_{3}q_{1}\right)),\\ \; & \psi =(2\left(q_{0}q_{3}+q_{1}q_{2}\right),1-2\left(q_{2}^{2}+q_{3}^{2}\right)). \end{array}$$

### 3.2. Environment Models

#### 3.2.1. International Geomagnetic Reference Field (IGRF)

The IGRF denotes the main geomagnetic field generated by internal sources, primarily inside the core of the Earth [30]. The absence of electric currents on the surface allows for computation of the Earth’s geomagnetic field as the negative gradient of a scalar potential, such that the magnetic field can be expressed as B=−δV. The potential function is represented by a finite series expansion in terms of Gauss coefficients, $g_{n}^{m}$ and $h_{n}^{m}$ [31]:

(16) $$\begin{array}{cc} B(r,\beta ,\alpha )=V(r,\beta ,\alpha ,t)= & a\sum_{n=1}^{N}\sum_{m=1}^{n}{\left(\frac{a}{r}\right)}^{n+1}\\ \; & \left[g_{n}^{m}\left(t\right)\cos m\alpha +h_{n}^{m}\left(t\right)\sin m\alpha \right]P_{n}^{m}\left(\cos\beta \right). \end{array}$$

Here, r,β,α,t are the radial distance from the Earth’s center, geocentric co-latitude, longitude and time, respectively. The variable *a* is the Earth’s radius. More information about the spherical harmonic coefficients can be found in [30]. The latest version in the series, IGRF-13, extends up to thirteen harmonic degrees.

#### 3.2.2. PSA Sun Position Algorithm

This paper utilizes the PSA algorithm to calculate the solar vector in the inertial frame due to its small computational footprint and high accuracy. It has been established that when applied over a period ranging from 2020 to 2050, the maximum error in angular deviation from the actual solar vector is only 35 arc-sec, with the algorithm maintaining its computational structure and simplicity [32]. The PSA algorithm’s inputs consist of the location’s latitude and longitude, along with the time specified in universal time (UT1), encompassing year, month, day, hours, minutes, and seconds. The sun vector is given by

(17) $$S=\left[\begin{array}{c} \sin\vartheta_{z}\sin\gamma \\ \sin\vartheta_{z}\cos\gamma \\ \cos\vartheta_{z} \end{array}\right]$$

where ϑ and γ are the zenith and azimuth angles. More information on how these angles are obtained can be found in [32].

### 3.3. Measurement Model

In this model, we assume that only zero-mean Gaussian white noise can corrupt the sensor measurements. The measurement model for the vector observations can be given as

(18) $$b_{i}=Ar_{i}+\mu_{i},$$

where b is the sun and magnetic field vector observations in the body frame, r can either be B or S modeled in the reference frame, μ is the Gaussian white noise, and A is the attitude matrix expressed in quaternions.

For gyroscopes, the inherent sensor bias introduces errors throughout the attitude determination process; therefore, these errors are also included in the state vector estimate. The gyroscope mathematical model presented in this paper is

(19) $${\hat{\omega }}_{b}=\omega_{b}+b+v,$$

where gyroscope noise and bias of a satellite are represented by b and v, respectively, while the true angular velocity of the satellite relative to the inertial frame is denoted by $\omega_{b}$.

### 3.4. Coordinate Systems

Several reference frames are used to describe the satellite’s attitude. This paper employs three reference frames, each of which is defined by its fixed element or direction, as well as by the location of its center or origin, that is, satellite body coordinate frame, orbit referenced coordinate frame and Earth centered inertial (ECI) coordinate frame. These coordinate frames have been well explained in [14].

## 4. Adaptive EKF

During extended eclipse phases, it was noted that the traditional extended Kalman filter (EKF) exhibited superior performance compared to other methods such as the singular value decomposition (SVD)-aided EKF. However, if the accuracy of the process and measurement noise covariance matrices is compromised, the EKF may produce substantial estimation errors, despite outperforming other algorithms in prolonged eclipses. As a result, there is a great need for developing an adaptive EKF that is compatible with inaccurate covariance matrices. Firstly, we discuss how each covariance matrix affects the performance of the extended Kalman filter (EKF). The process noise covariance matrix $Q_{k}$ can cause inaccurate prediction of the error covariance matrix $P_{k+1}$ in (7), which directly affects the computation of the Kalman gain K as shown in (8). Finally, the optimal estimate $x_{k}$ and the estimated error covariance matrix are derived from the incorrect $P_{k+1}$ and K as seen in (9) and (10), respectively. Additionally, it is clear that an imprecise measurement noise covariance matrix $R_{k}$ can lead to an imprecise Kalman gain, resulting in inadequate state estimation. It is apparent that P and R directly affect the state estimate as compared to Q. In this paper, a P and R adaptive filter is proposed for attitude estimation. The filter performance will be compared to the SVD-aided EKF in the sun and eclipse phases, which performs better than the conventional EKF in attitude estimation.

### 4.1. Expectation Maximization

There is usually inadequate information for training Bayesian networks; for example, it is difficult to accurately determine noise covariance matrices in a Kalman filter [33]. Such latent parameters can explicitly be estimated using the expectation maximization (EM) algorithm, which is an iterative algorithm used in unsupervised learning. The EM algorithm is an approach used to find the maximum likelihood (ML) estimates of the latent variables in a statistical method. It is used to find the maximum likelihood estimate of the parameters, given the observed data. The algorithm consists of two steps: the expectation step, where the expectation of the log-likelihood function is calculated, given the current estimates of the parameters, and the maximization step (M-step), where the parameters are updated to maximize the expected log-likelihood [34]. The algorithm iterates between these two steps until convergence, at which point the parameters are considered to be the maximum likelihood estimates. The goal of this paper is to use the joint log-likelihood function to estimate inaccurate noise covariance matrices $P_{k}$ and $R_{k}$ based on the available measurements $z_{1:k}$ (i.e., measurements taken from time 1 to *k*) and the unknown state vector $x_{k}$, that is,

(20) $$L_{\sigma_{k}}\left(x_{k},z_{k}\right)=\log p_{\sigma_{k}}\left(x_{k},z_{k}\right),$$

where $\sigma_{k}=\left[P_{k},R_{k}\right]$. The joint log-likelihood function contains the natural logarithm function log and the probability density function *p*, which depends on the parameter σ. To obtain an approximate maximum likelihood (ML) solution for the parameter $\sigma_{k}$, we employ the expectation maximization (EM) algorithm, which maximizes the joint log-likelihood function, such that

(21) $$\begin{array}{cc} {\hat{\sigma }}_{k} & =\arg\max L_{\sigma_{k}}\left(x_{k},z_{k}\right)\\ & ≜\arg\max\log p_{\sigma_{k}}\left(x_{k},z_{k}\right), \end{array}$$

where ${\hat{\sigma }}_{k}$ is the ML estimate of $\sigma_{k}$. It is impossible to solve the complete data log-likelihood function $L_{\sigma_{k}}\left(x_{k},z_{k}\right)$ due to the unavailable $x_{k}$. The EM algorithm addresses the problem by approximating the joint likelihood function in (21) as its minimum variance estimate $Q(\sigma_{k},\sigma_{k}^{i})$, such that [35,36]

(22) $$\begin{array}{cc} Q(\sigma_{k},\sigma_{k}^{i})≜ & E_{\sigma_{k^{i}}}\left[L_{\sigma_{k}}\left(x_{k},z_{k}\right)|z_{k}\right]\\ = & \int L_{\sigma_{k}}\left(x_{k},z_{k}\right)p_{\sigma_{k}}\left(x_{k}|z_{k}\right)dx_{k}, \end{array}$$

where $\sigma_{k}^{i}$ is an approximation of ${\hat{\sigma }}_{k}$ at the *i*th step.

The EM algorithm takes advantage of this property to generate a series of values $\sigma_{k}$, where k=1,2,⋯, with the aim of successively improving the accuracy of the maximum likelihood (ML) estimate. The algorithm is summarized in Algorithm 1. During the E-step, the complete data log-likelihood’s expectation is evaluated, which relies on the current estimates $\sigma_{k}^{i}$ and measurements $z_{1:k}$. Following this, the M-step maximizes the computed $Q(\sigma_{k},\sigma_{k}^{i})$ utilizing an arithmetic technique.
**Algorithm 1** The expectation maximization algorithm1:Initialization: $\sigma_{k}$2:E-step:Compute $Q(\sigma_{k},\sigma_{k}^{i})$3:Maximization:Calculate, $\sigma_{k+1}=\arg\max Q\left(\sigma_{k},\sigma_{k}^{i}\right)$4:If convergence has not been reached, proceed to update the variable *k* by incrementing it by 1, and return to step 2.

#### 4.1.1. E Step

The EM algorithm approximates the joint likelihood function as its minimum variance estimate as shown in (22). To determine the minimum variance estimate, we compute the joint log likelihood $\log p\sigma_{k}\left(x_{k},z_{1:k}\right)$ and the second posterior PDF $p_{\sigma_{k}}\left(x_{k}|z_{k}\right)$. Firstly, the joint log likelihood in (20) is written as the product of the conditional likelihood, such that

(23) $$\begin{array}{cc} p_{\sigma_{k}}\left(x_{k},z_{1:k}\right) & =p_{\sigma_{k}}\left(z_{k}|x_{k},z_{1:k}\right)p_{\sigma_{k}}\left(x_{k}|z_{1:k}\right)p\left(z_{1:k}\right)\\ & =p_{\sigma_{k}}\left(z_{k}|x_{k}\right)p_{\sigma_{k}}\left(x_{k}|z_{1:k}\right)p\left(z_{1:k}\right) \end{array}$$

where $p_{\sigma_{k}}\left(z_{k}|x_{k},z_{1:k}\right)=p_{\sigma_{k}}\left(z_{k}|x_{k}\right)$, since the expected measurement $z_{k}$ is only dependent on state $x_{k}$ as shown in (5). The probability distribution $p(z_{1:k})$ of the sensor measurements is typically not dependent on the current state error and measurement noise covariance matrix $\sigma_{k}$. This is because the measurements are generally assumed to be independent of both the state error covariance matrix and the measurement noise covariance matrix, which together determine the joint distribution of the state estimates and the measurements [36]. In the EKF model, the predicted PDF $p_{\sigma_{k}}\left(x_{k}|z_{1:k}\right)$ is approximated as Gaussian, that is,

(24) $$p_{\sigma_{k}}\left(x_{k}|z_{1:k}\right)=N\left(x_{k};x_{k+1},P_{k-1}\right).$$

The symbol N(.;μ,∑) represents the Gaussian probability density function (PDF) with mean vector μ and covariance matrix ∑, and $x_{k+1}$ is obtained using Equation (6). At this stage, $P_{k-1}$ is considered inaccurate because of $Q_{k}$. The likelihood PDF of the measurement model in (7) is given by

(25) $$p_{\sigma_{k}}\left(z_{k}|x_{k}\right)=N\left(z_{k};h\left(x_{k+1},k\right),R_{k}\right).$$

The joint log-likelihood function can be computed using (23)–(25). The logarithm of the normal distribution $N(z_{k};h\left(x_{k+1},k\right),R_{k})$ is:

(26) $$\log N\left(z_{k};h\left(x_{k+1},k\right),R_{k}\right)=-0.5\log\left|R_{k}\right|-0.5{\left[z_{k}-h\left(x_{k+1},k\right)\right]}^{T}R^{-1}\left[z_{k}-h\left(x_{k+1},k\right)\right].$$

The logarithm of the normal distribution $N(x_{k};x_{k+1},P_{k-1})$ is:

(27) $$\log N\left(x_{k};x_{k+1},P_{k-1}\right)=-0.5\log\left|P_{k+1}\right|-0.5{\left(x_{k}-x_{k+1}\right)}^{T}P_{k+1}^{-1}\left(x_{k}-x_{k+1}\right).$$

Putting everything together:

(28) $$\begin{array}{cc} \log p_{\sigma_{k}}\left(x_{k},z_{1:k}\right)= & -0.5|R_{k}|-0.5{\left[z_{k}-h\left(x_{k+1},k\right)\right]}^{T}R^{-1}\left[z_{k}-h\left(x_{k+1},k\right)\right]\\ & -0.5|P_{k+1}|-0.5{\left(x_{k}-x_{k+1}\right)}^{T}P_{k+1}^{-1}\left(x_{k}-x_{k+1}\right)+d_{\sigma_{k}}, \end{array}$$

where |.| is the determinant operation of a matrix. After determining the joint likelihood in (28), the posterior PDF is computed. At the $i+1^{th}$ step, the state vector $x_{k}^{i}$ estimate and corresponding state estimation error covariance matrix $P_{k}^{i}$ have been calculated. From here, the nonlinear measurement function is linearized using the intermediate state estimate $x_{k}^{i}$, that is,

(29) $$h\left(x_{k},k\right)=h\left({\hat{x}}_{k}^{i},k\right)+H_{k}^{i}\left(x_{k+1}-{\hat{x}}_{k}^{i}\right),$$

where $H_{k}^{i}$ is the Jacobian matrix of the measurement function at $x_{k}^{i}$. The second posterior PDF $p_{\sigma_{k}^{i}}\left(x_{k}|z_{1:k}\right)$ is approximated as Gaussian by using a measurement update of the EKF, such that

(30) $$p_{\sigma_{k}^{i}}\left(x_{k}|z_{1:k}\right)=N\left(x_{k};x_{k}^{i+1},P_{k}^{i+1}\right)$$

where $P_{k}^{i+1}$ and $x_{k}^{i+1}$ are obtained using $P_{k}^{i}$ and $x_{k}^{i}$, respectively, given as:

(31) $$K_{k}^{i+1}=P_{k+1}^{i}{\left(H_{k}^{i}\right)}^{T}{\left[H_{k}^{i}P_{k+1}^{i}{\left(H_{k}^{i}\right)}^{T}+R_{k}^{i}\right]}^{-1}$$

(32) $$x_{k}^{i+1}=x_{k+1}+K_{k}^{i+1}\left[z_{k}-\left(h\left({\hat{x}}_{k}^{i},k\right)+H_{k}^{i}\left(x_{k+1}-{\hat{x}}_{k}^{i}\right)\right)\right]$$

(33) $$P_{k}^{i+1}=P_{k+1}^{i}-K_{k}^{i+1}H_{k}^{i}P_{k+1}^{i}$$

To obtain the minimum variance estimate, Equations (28) and (30) are substituted into Equation (22). The resulting expression is

(34) $$\begin{array}{cc} \; & Q\left(\sigma_{k},\sigma_{k}^{i}\right)=-0.5\left|R_{k}\right|-0.5\mathrm{tr}\left(A_{k}R_{k}^{-1}\right)\\ & -0.5|P_{k+1}|-0.5\mathrm{tr}\left(B_{k}P_{k+1}^{-1}\right)+d_{\sigma_{k}} \end{array}$$

where tr is the trace of a matrix. Matrices $A_{k}$ and $B_{k}$ are given by

(35) $$A_{k}=\int \left[z_{k}-h\left(x_{k+1}\right)\right]{\left[z_{k}-h\left(x_{k+1}\right)\right]}^{T}N\left(x_{k};x_{k}^{i+1},P_{k}^{i+1}\right)dx_{k}$$

(36) $$B_{k}=\int \left(x_{k}-x_{k+1}\right){\left(x_{k}-x_{k+1}\right)}^{T}N\left(x_{k};x_{k}^{i+1},P_{k}^{i+1}\right)dx_{k}.$$

$A_{k}$ represents the squared Mahalanobis distance between the prediction and the observation, and $B_{k}$ can be seen as an intermediate step in the calculation of the expected value. The measurement is further linearized at $x_{k}^{i+1}$ such that

(37) $$h\left(x_{k},k\right)=h\left({\hat{x}}_{k}^{i+1},k\right)+H_{k}^{i+1}\left(x_{k+1}-{\hat{x}}_{k}^{i+1}\right),$$

Equation (37) is substituted in (35); thus, $A_{k}$ is computed as

(38) $$A_{k}=\left[z_{k}-h\left({\hat{x}}_{k}^{i+1},k\right)\right]\left[z_{k}-h\left({\hat{x}}_{k}^{i+1},k\right)\right){]}^{T}+H_{k}^{i+1}P_{k}^{i+1}{\left(H_{k}^{i+1}\right)}^{T}.$$

From there, $B_{k}$ can be computed as

(39) $$B_{k}=P_{k}^{i+1}+\left({\hat{x}}_{k}^{i+1}-x_{k+1}\right){\left({\hat{x}}_{k}^{i+1}-x_{k+1}\right)}^{T}$$

#### 4.1.2. M Step

The M-step entails maximizing $Q(\sigma_{k},\sigma_{k}^{i})$ regarding $\sigma_{k}$, such that

(40) $$\sigma_{k}^{i+1}\approx \arg\max Q\left(\sigma_{k},\sigma_{k}^{i}\right).$$

At the maximum point, $\sigma_{k}^{i+1}$ satisfies

(41) $$\frac{\delta Q(\sigma_{k},\sigma_{k}^{i})}{\delta \sigma_{k}}{|}_{\sigma_{k}=\sigma_{k}^{i+1}}=0.$$

Since $\sigma_{k}=[P_{k-1},R_{k}]$, (41) becomes a partial derivative problem, that is, $\frac{\delta Q(\sigma_{k},\sigma_{k}^{i})}{\delta P_{k-1}}$ and $\frac{\delta Q(\sigma_{k},\sigma_{k}^{i})}{\delta R_{k}}$. The partial derivative can be calculated by exploiting (34) as

(42) $$\frac{\delta Q(\sigma_{k},\sigma_{k}^{i})}{\delta P_{k-1}}=-0.5P_{k-1}^{-1}+0.5P_{k-1}^{-1}B_{k}P_{k-1}^{-1}$$

(43) $$\frac{\delta Q(\sigma_{k},\sigma_{k}^{i})}{\delta R_{k}}=-0.5R_{k}{-}^{-1}+0.5R_{k}^{-1}B_{k}R_{k}^{-1}$$

where $A_{k}$ and $B_{k}$ are given by (35) and (36), respectively. Substituting (42) and (43) in (41) yields

(44) $$-0.5{\left(P_{k-1}^{i+1}\right)}^{-1}+{\left(P_{k-1}^{i+1}\right)}^{-1}B_{k}{\left(P_{k-1}^{i+1}\right)}^{-1}=0$$

(45) $$-0.5{\left(R_{k}^{i+1}\right)}^{-1}+{\left(R_{k}^{i+1}\right)}^{-1}A_{k}{\left(R_{k}^{i+1}\right)}^{-1}=0;$$

therefore, the error and measurement noise covariance matrices are defined as $P_{k-1}^{i+1}=B_{k}$ and $R_{k}^{i+1}=A_{k}$, respectively.

### 4.2. P and R Adaptive EKF

The proposed adaptive extended Kalman filter (EKF) algorithm in Algorithm 2 involves two primary steps: the time update and the iterated measurement update. In the time update, the predicted state vector $x_{k+1}$ and the nominal predicted state error covariance matrix $P_{k+1}$ are calculated using Equations (6) and (7), respectively. The algorithm requires several inputs, including the initial state vector $x_{k-1}$, state error covariance matrix $P_{k-1}$, measurement covariance matrix $R_{k}$, process noise covariance matrix $Q_{k}$, and the number of iterated measurements *N*. The prediction step only computes the state vector and error covariance matrix, considering the system model kinematics and the previous state error covariance matrix. The nominal predicted error covariance matrix $P_{k+1}$ serves as an appropriate initial value for $P_{k+1}^{0}$, as it includes the information on the state transition matrix F, process noise covariance matrix Q, and the previous estimation error covariance matrix $P_{k-1}$. The **for** loop estimates the optimal state vector $x_{k}$, predicted error covariance matrix $P_{k}$, and measurement noise covariance matrix $R_{k}$ in an adaptive manner.
**Algorithm 2  **P and R adaptive EKF for attitude estimation.**Inputs**: $x_{k-1},P_{k-1},{\tilde{R}}_{k},Q_{k},B_{b},S_{b},\omega ,N,t$     **Prediction Step**:1.$x_{k+1}=F_{k}x_{k-1}+B\omega$2.$P_{k+1}=F_{k}P_{k-1}F_{k}^{T}+Q_{k}$**Measurement update**3.Initialize at $i=0,P_{k+1}^{\left(0\right)}=P_{k+1},$   $R_{k}^{0}={\tilde{R}}_{k},x_{k+1}^{0}=x_{k+1}$**for** i=0:N−14.Compute the Jacobian matrix of $h(x_{k+1},k)$ to find $H_{k}^{i}$5.$K_{k}^{i}=\frac{P_{k+1}^{i}H_{k}^{iT}}{H_{k}^{i}P_{k+1}^{i}{\left(H_{k}^{i}\right)}^{T}+R_{k}^{i}}$6.$\left[\begin{array}{cc} B_{b} & S_{b} \end{array}\right]$7.$x_{k}^{i}=x_{k+1}+K_{k}^{i}\left(z_{k}-h\left(x\right)-H_{k}^{i}x_{k+1}\right)$8.$P_{k}^{i}=P_{k+1}-K_{k}^{i}H_{k}^{i}P_{k+1}$9.Compute Jacobian matrix $H_{k}^{i=1}$ with updated states estimates $x_{k}^{i}$10.$A_{k}=\left[z_{k}-h\left(x\right)\right]{\left[z_{k}-H_{k}^{i}x_{k+1}\right]}^{T}+H_{k}^{i+1}P_{k}^{i}{\left(H_{k}^{i+1}\right)}^{T}$11.$B_{k}=P_{k}^{i}+\left(x_{k}^{i}-x_{k+1}\right){\left(x_{k}^{i}-x_{k+1}\right)}^{T}$12.$P_{k}^{i+1}=B_{k},R_{k}^{i+1}=A_{k}$**end for**13.${\hat{P}}_{k}=P_{k}^{i+1},{\hat{R}}_{k}=R_{k}^{i+1}$**outputs:** $x_{k},{\hat{P}}_{k},{\hat{R}}_{k}$

## 5. Results and Analysis

In the simulations, a CubeSat in a low Earth orbit is considered, with a principal moment of inertia of $J=\mathrm{diag}\left[\begin{array}{ccc} 0.0071 & 0.0035 & 0.0035 \end{array}\right]\;$kg.m${}^{2}$. The spacecraft is assumed to be tumbling, and its initial state is arbitrarily chosen as $x_{0}={\left[0.03\;\mathrm{rad}\;-0.001\;\mathrm{rad}\;0.002\;\mathrm{rad}\;0.08\;\mathrm{rad}/s\;-0.02\;\mathrm{rad}/s\;0.03\;\mathrm{rad}/s\right]}^{T}$. The orbit parameters, satellite kinematics, and sensor and environmental models are used to validate the proposed algorithm for attitude estimation. In this study, the sun sensor, magnetometer, and gyroscope are used as attitude sensors. The local magnetic vector is computed using the international geomagnetic reference field model, while a sun position algorithm is used to extract the local reference sun vector. The orbit used in this paper is circular with an inclination of 0${}^{\circ }$ at an altitude of 600 km. The simulation epoch is 2021.01.01, UTC 00:00 with a time-step of 1 s.

The first part of this section is devoted to analyzing the performance of SVD-EKF and proposed AEKF using the Big O notation, the second part analyzes the algorithms during sun phase on the orbit. The third part analyzes the performance of the two algorithms during the eclipse phase. The simultaneous P and R adaptive extended Kalman filter is referred to as AEKF in this study. The evaluation of the estimation accuracy for the measurement noise covariance matrix and predicted error covariance matrix was carried out using the SRNFN and ASRNFN metrics, which were chosen as the error measures. These metrics provide information about the magnitude of the errors in the estimated matrices by measuring the difference between the estimated and reference values, and they are defined as

(46) $$\mathrm{SRNFN}={\left(\frac{1}{n^{2}}{P_{k+1}-P_{r,k+1}}^{2}\right)}^{\frac{1}{4}},$$

(47) $$\mathrm{ASRNFN}=\frac{1}{T}\sum_{k=1}^{T}{\left(\frac{1}{n^{2}}{P_{k+1}-P_{r,k+1}}^{2}\right)}^{\frac{1}{4}}.$$

The predicted error covariance at time *k* is represented by $P_{k+1}$, while $P_{r,k+1}$ refers to the reference value of the predicted error covariance matrix at that same time. Similarly, the formulas used for calculating SRNFN and ASRNFN for the predicted error covariance matrix are also applied to the measurement noise covariance matrix. The SRNFN is a metric that measures the similarity between two matrices. The SRNFN is widely used in the field of computer vision and image processing as a measure of the similarity between two images [37]. The Averaged Square Root of Normalized Frobenius Norm (ASRNFN) is an extension of the SRNFN, which is used to measure the similarity between multiple matrices. The ASRNFN is calculated as the average of the SRNFN between each pair of matrices [38].

### 5.1. Complexity of Algorithms

The big O notation is a mathematical notation used to analyze and describe the performance or complexity of algorithms. It provides a framework to express how the runtime or resource usage of an algorithm grows as the input size increases. The notation is represented as O(f(n)), where “O” stands for order of magnitude and “f(n)” represents a function that describes the algorithm’s growth rate. The function “f(n)” typically represents the number of operations or resource usage as a function of the input size “n”. The purpose of the big O notation is to provide a simplified approximation of the algorithm’s complexity, ignoring constant factors and lower-order terms. By analyzing the big O complexity of algorithms, researchers and developers can make informed decisions about algorithm selection, understand the trade-offs between runtime and resources, and identify areas for optimization. It provides a standardized framework for reasoning about algorithmic efficiency and assists in designing more efficient algorithms to effectively solve computational problems.

- SVD-aided EKF
  The operations of predicting the state and error covariance matrix involve matrix multiplication, which has a time complexity of $O(n^{3})$, where n is the size of the matrices involved (in this case, 7 × 7 matrices). The computation of B involves multiplication and addition operations with matrices and vectors. Assuming that the input vectors have dimensions 3 × 1, the complexity of this section is O($3^{2}$) or O(1). The SVD operation performed on the matrix B has a complexity of O($min(m^{2}n,mn^{2})$), where *m* and *n* are the dimensions of matrix B. Since B has dimensions 3x3, the complexity is O($3^{3}$) or O(1). In conclusion, the overall complexity of the SVD-aided EKF can be approximated as $O(n^{3})$.
- Proposed adaptive filter
  The prediction part of the algorithm is similar to that of the SVD-aided EKF, and therefore, the complexity is O($n^{3}$). The loop iterates N−1 times. The operations such as calculating gain involve matrix multiplication, which has a time complexity of O($N\times n^{3}$), where n is the size of the matrices involved (in this case, 6 × 7 and 7 × 7 matrices). The remaining operations in the loop, such as matrix additions and subtractions, have a constant time complexity, O(1). Overall, the time complexity is given by O(N × O($n^{3}$)) = O($n^{3}$).

Although both the SVD-aided extended Kalman filter (EKF) and the proposed adaptive EKF algorithms have similar complexities in terms of their big O notation, the complexity analysis only provides an approximation of the computational requirements. The actual runtime performance of the algorithms may differ based on various factors, such as the specific implementation details and the size of the input data.

### 5.2. Sun Phase

Firstly, we compare the performance of the SVD-EKF and the proposed AEKF during sun phases. During the sun phase or normal operation mode, the filter depends on the sun sensor and magnetometer measurements for measurement update. The proposed algorithm is affected by the number of iterated measurements *N*: the more that is iterated, the more accurate the state estimates; however, this means an increased computational time. For these simulations, N=5. Table 1 shows the root mean square error of the attitude angles for both filters considered in this study. It is evident that both filters perform reasonably well under the sun mode, as all sensor measurements are available.

**Figure 2:**
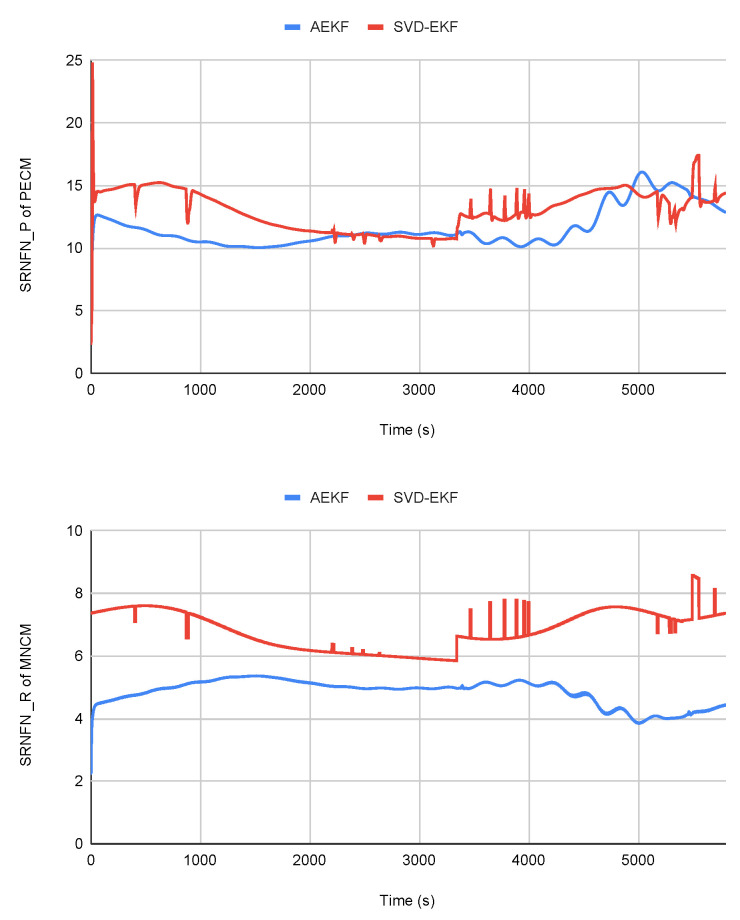
SRNFN${}_{P}$ and SRNFN${}_{R}$ of the two filters during sun phase.

Table 2 shows the single-step run time for the two algorithms. It shows that the proposed algorithm has more computational time as compared to the SVD-EKF. Even though the computational load is higher by only a small margin, the accuracy of the proposed algorithm is significant. To demonstrate the efficacy of the proposed algorithm, we calculated the SRNFN and ASRNFN values of the predicted error and measurement noise covariance matrices, which are useful in analyzing the convergence of these matrices. An analysis of the results presented in Figure 2 and Table 3 indicate that the proposed algorithm outperforms the SVD-EKF algorithm, as evidenced by its lower SRNFN and ASRNFN values.

### 5.3. Eclipse Phase

According to the study in [20], satellites in low Earth orbit are in the eclipse phase for approximately 28 min. During this phase, the attitude-determination system depends solely on magnetometer measurements. Table 4 displays the root mean square error of the SVD-EKF method and AEKF. While the proposed algorithm’s error increased slightly, it still performs significantly better than the SVD-aided EKF. Table 4 confirms that the gyroscope bias increased during the eclipse phase, as compared to the values in Table 1. This increase in bias is likely due to the temperature changes that occur during the eclipse phase. Although modern gyroscopes are designed to minimize temperature-related errors and often include temperature-compensation features, the effects of temperature changes cannot be eliminated. Therefore, it is crucial to consider the potential impact of temperature changes on gyroscope bias when designing and implementing attitude-determination systems for satellites.

**Figure 3:**
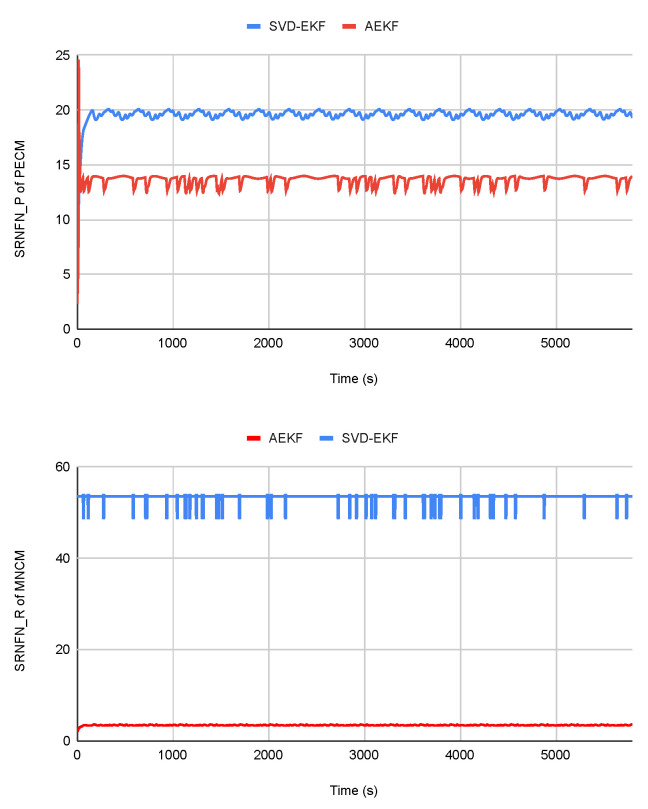
SRNFN${}_{P}$ and SRNFN${}_{R}$ of the two filters during eclipse phase.

The inferior estimation accuracy of the SVD-EKF algorithm during the eclipse phase is likely due to the sensitivity of the SVD algorithm, which requires at least two vector measurements to compute attitude. Despite relying solely on magnetometer measurements, the proposed algorithm performs reasonably well. Furthermore, Figure 3 and Table 5 show that the proposed algorithm has a smaller SRNFN and ASRNFN than the SVD-EKF even in the eclipse phase. The noise during the eclipse phase affects the measurements used to estimate the measurement noise and state covariance matrices; therefore, the resulting matrices are less accurate or less precise.

## 6. Conclusions

This paper presents the design and numerical analysis of an error and measurement noise covariance matrix-adaptive extended Kalman filter algorithm based on the expectation maximization method. A novel attitude-estimation algorithm was created utilizing an adaptive filter approach. The performance of this newly developed algorithm, as well as a state-of-the-art existing algorithm, was evaluated through application on CubeSat attitude estimation in both the eclipse and sun phases of the orbit. During both phases, the proposed adaptive EKF outperforms the existing algorithm, being the SVD-EKF. The SVD algorithm requires a minimum of two vector measurements and associated reference models to function optimally. However, during the eclipse phase, only magnetometer measurements are available, which causes the performance of the SVD-aided algorithm to deteriorate. Based on the simulation findings outlined in the paper, the proposed algorithm demonstrated significantly improved accuracy in attitude estimation compared to SVD-aided EKF algorithms. However, it was also observed that the proposed algorithm has marginally higher computational complexity.

To ensure the accuracy and reliability of the proposed algorithm in various conditions and applications, it is necessary to conduct rigorous validation and verification through both simulations and real-world tests. This will help to confirm the effectiveness of the algorithm and to identify any potential limitations or areas for improvement. Furthermore, the proposed algorithm can be optimized to improve its performance and to reduce computational complexity. This could involve refining the algorithm’s underlying mathematical models and algorithms, as well as optimizing its implementation and hardware requirements. By doing so, the algorithm could potentially be made more efficient and practical for use in a wider range of systems and applications.

Given the promising outcomes achieved by the proposed algorithm, future research will primarily focus on optimizing the algorithm to significantly decrease its overall complexity. This optimization endeavor will involve a comprehensive exploration of various techniques and methodologies aimed at streamlining the algorithm’s computational demands while maintaining its accuracy and performance. To accomplish this, each component of the algorithm will be meticulously examined to identify potential areas for improvement. Strategies such as algorithmic modifications, efficient data structures, and parallel computing techniques will be employed to alleviate the computational burden. The objective of these efforts is to strike a balance between complexity reduction and algorithmic efficiency, enabling the algorithm to be more practical and applicable in real-world scenarios with limited resources.

## Figures and Tables

**Table 1:** Comparison of the root mean square errors of the SVD-EKF and proposed algorithm during the sun phase.

RMSE	SVD-EKF	AEKF
ϕ ${(}^{\circ })$	0.2146	0.0042
θ ${(}^{\circ })$	0.2220	0.0015
ψ ${(}^{\circ })$	0.2472	0.0024
$\omega_{\phi }$ ${(}^{\circ }/s)$	3.6076 × 10${}^{-6}$	4.4296 × 10${}^{-8}$
$\omega_{\theta }$ ${(}^{\circ }/s)$	7.7722 × 10${}^{-6}$	8.8530 × 10${}^{-8}$
$\omega_{\psi }$ ${(}^{\circ }/s)$	8.8545 × 10${}^{-6}$	1.7761 × 10${}^{-8}$

**Table 2:** Single-step run execution of the proposed algorithm and SVD-EKF.

Filters	SVD-EKF	AEKF
Time (s)	7.94 × $10^{-4}$	8.46 × $10^{-4}$

**Table 3:** Evaluation of the proposed algorithm and SVD-Aided EKF in sunlit phase using ASRNFN${}_{P}$ and ASRNFN${}_{R}$.

Filters	SVD-EKF	AEKF
ASRNFN ${}_{P}$	13.0078	11.6005
ASRNFN ${}_{R}$	47.1554	4.8422

**Table 4:** Comparison of the root mean square errors of the SVD-EKF and proposed algorithm during eclipse phase.

RMSE	SVD-EKF	AEKF
ϕ ${(}^{\circ })$	1.7126	0.1094
θ ${(}^{\circ })$	12.6611	0.1388
ψ ${(}^{\circ })$	6.9083	0.3137
$\omega_{\phi }$ ${(}^{\circ }/s)$	7.9837 × 10${}^{-5}$	1.3449 × 10${}^{-7}$
$\omega_{\theta }$ ${(}^{\circ }/s)$	1.3499 × 10${}^{-5}$	6.8046 × 10${}^{-8}$
$\omega_{\psi }$ ${(}^{\circ }/s)$	1.9008 × 10${}^{-4}$	1.8077 × 10${}^{-8}$

**Table 5:** Evaluation of the proposed algorithm and SVD-aided EKF in eclipse phase using ASRNFN${}_{P}$ and ASRNFN${}_{R}$.

Filters	SVD-EKF	AEKF
ASRNFN ${}_{P}$	19.5559	13.6083
ASRNFN ${}_{R}$	53.2372	3.4397

## Data Availability

No new data were created or analyzed in this study. Data sharing is not applicable to this article.
